# Performance and determinants of routine immunization coverage within the context of intensive polio eradication activities in Uttar Pradesh, India: Social Mobilization Network (SM Net) and Core Group Polio Project (CGPP)

**DOI:** 10.1186/1472-698X-13-25

**Published:** 2013-05-16

**Authors:** William M Weiss, Manojkumar Choudhary, Roma Solomon

**Affiliations:** 1 Department of International Health, Johns Hopkins Bloomberg School of Public Health, 615 North Wolfe Street, Suite E8132, Baltimore, MD, 21205, USA; 2 CORE Group Polio Project – India, 303, Radisson Suites, Commercial Tower, B-Block, Sushant Lok-I, Gurgaon, Haryana, 122002, India

## Abstract

**Background:**

Studies that have looked at the effect of polio eradication efforts in India on routine immunization programs have provided mixed findings. One polio eradication project, funded by US Agency for International Development (USAID) and carried out by the CORE Group Polio Project (CGPP) in the state of Uttar Pradesh of India, has included the strengthening of routine immunization systems as a core part of its polio eradication strategy. This paper explores the performance of routine immunization services in the CGPP intervention areas concurrent with intensive polio eradication activities. The paper also explores determinants of routine immunization performance such as caretaker characteristics and CGPP activities to strengthen routine immunization services.

**Methods:**

We conduct secondary data analysis of the latest project household immunization survey in 2011 and compare these findings to reports of past surveys in the CGPP program area and at the Uttar Pradesh state level (as measured by children’s receipt of DPT vaccinations). This is done to judge if there is any evidence that routine immunization services are being disrupted. We also model characteristics of survey respondents and respondents’ exposure to CGPP, communication activities against their children’s receipt of key vaccinations in order to identify determinants of routine immunization coverage.

**Results:**

Routine immunization coverage has increased between the first survey (2005 for state level estimates, 2008 for the CGPP program) and the latest (2011 for both state level and CGPP areas), as measured by children’s receipt of DPT vaccination. This increase occurred concurrent with polio eradication efforts intensive enough to result in interruption of transmission. In addition, a mothers’ exposure to specific communication materials, her religion and education were associated with whether or not her children receive one or more doses of DPT.

**Conclusions:**

A limitation of the analysis is the absence of a controlled comparison. It is possible routine immunization coverage would have increased even more in the absence of polio eradication efforts. At the same time, however, there is no evidence that routine immunization services were disrupted by polio eradication efforts. Targeted health communications are helpful in improving routine immunization performance. Strategies to address other determinants of routine immunization, such as religion and education, are also needed to maximize coverage.

## Background

In 1988, the estimated number of wild poliovirus cases worldwide was 350,000 [1]. However, by the end of 2012, the total number fell to 223 [2]. As of 6 March 2013, the total number of 2013 wild polio cases worldwide is nine compared to 22 by this date in 2012; all cases (9/9) are in the remaining three endemic countries of Afghanistan, Nigeria and Pakistan [2]. There have been no reported cases of wild poliovirus in India since January 2011 [3]. This is a remarkable accomplishment, especially in India.

Questions have arisen as to how the tremendous polio eradication effort in India may have affected routine immunization programs for polio and non-polio antigens. Loevinsohn et al. (2002) reviewed several studies and found no association globally between polio eradication efforts and a decrease in funding for routine immunization or a decrease in routine immunization coverage [4], but raised concerns about shifting the time of primary health workers from duties such as routine immunization to support polio eradication campaigns. Yadav et al. (2009) found that polio eradication efforts in India had led to interruptions in primary health care services [5]. Bonu et al. (2003) found an association between polio eradication efforts in Northern India and an increase in the first dose of polio and non-polio routine immunization vaccines, but found no increase in receipt of 2^nd^ and 3^rd^ doses--- indicating little synergy between eradication and routine immunization efforts [6]. The importance of improving very poor routine immunization coverage levels in India alongside intensive polio eradication efforts, however, has been argued as critical for eradication (e.g., helping prevent importation of the polio virus), for equity purposes, and for health systems development [7-9]. Since 1996, the US Agency for International Development (USAID) has provided support to the global polio eradication effort and has included the strengthening of routine immunization systems as a core part of its strategy [10]. One USAID-funded polio eradication project that follows this part of the strategy in India is the CORE Group Polio Project.

The CORE Group is an umbrella organization of non-governmental organizations (NGOs) that collaborate on international health and development programs [11]. In India, the CORE Group Polio Project (CGPP) works across twelve districts in the state of Uttar Pradesh (UP). CGPP is a collaboration of the following NGOs: Adventist Development & Relief Agency (ADRA) India, Project Concern International (PCI) and Catholic Relief Services (CRS), as well as their ten local NGO partners. CGPP is a member of the Social Mobilization Network (SM Net) in India that also includes Unicef, Rotary, the Indian Government’s and WHO’s National Polio Surveillance Project (NPSP) as partners. The SM Net was created in 2003 to work in the northern state of Uttar Pradesh (UP). The SM Net supports polio eradication with the following efforts: identifying high-risk areas and working with underserved communities in planning, implementing and monitoring social mobilization and other immunization activities in those high-risk areas. The primary effort of the SM Net is carried out by a three-level network of community mobilizers (community level, block level, and district level) [12].

The Community Mobilization Coordinator (CMC) interacts with families and community members at the village level. As the backbone of the SM Net, s/he is assigned responsibility for mobilizing about 500 households in either a rural or an urban area, and keeps records of the immunization status of all children less than five years of age in those households. CMC areas are groups of communities in a block where the SM Net deploys CMCs. The SM Net selects these communities for additional social mobilization efforts based on past communication and operational challenges for immunizing children. Most of the CMCs are deployed in areas designated as High Risk Areas (HRAs). Jointly with key partners (Unicef, MOH and CGPP), NPSP defines the criteria for HRAs; these criteria are reviewed periodically and modified. The most recent criteria for HRAs take into account the following information: the number of wild polio virus (P1) cases during low transmission seasons since 2003; the presence of high risk groups (slum dwellers/nomads); the number of acute flaccid paralysis cases that were compatible with polio in last two years; if 40% or more of the population is Muslim; and, the percent of households that have unvaccinated children (called X houses). Once an area is identified as an HRA, the SM Net arranges for CMCs to work there. A CMC has to be 18 years or more, preferably female and from the same community. The partnership periodically revises the areas designated as an HRA. See Weiss et al. (2011 & 2013) for more details about the polio eradication activities of the CGPP [12,13].

In addition to other intensive polio eradication activities such as social mobilization for mass polio vaccination campaigns, CGPP India supports routine immunization (RI) since high RI coverage forms one of the main pillars of polio eradication. Each CMC is responsible for ensuring that all children in her allocated households are given all childhood vaccines, in addition to oral polio vaccine (OPV). She does this by doing home visits to track all eligible children and explain the importance of RI to the mothers. Just a day before the RI session, she distributes RI invitation slips to the mothers of eligible children. She also explains the importance of a Government RI Card that is completed by the auxiliary nurse midwife (ANM) after each immunization is given. She gives a specially designed Congratulatory Card to all families with newborns; this card has key health messages in an illustrative format. Apart from this, RI Camps are held in high risk areas where it is the CMC’s responsibility to see that all missed children are given the appropriate vaccine. Specific activities of CMCs that support RI are described below.

### Interpersonal communication (IPC) meetings

The interpersonal communication (IPC) meeting with mothers and caregivers---especially with those who express resistance to polio vaccination---is a major CMC activity during the interval between mass vaccination campaigns for polio eradication. The mass campaigns are also called supplemental immunization activities or SIAs. The purpose of an IPC meeting is to address misconceptions, rumors and fear through face-to-face dialogue. During IPC meetings, the CMC shares information about polio: how the virus is transmitted, and how transmission can be prevented. S/he also promotes routine immunization of all antigens, as well as polio immunization during each SIA. We expect that successful IPC meetings will lead to an increase in both routine EPI vaccines and supplemental polio vaccines.

### Mother’s meetings

Apart from IPC meetings with the mothers, the CMC also conducts meetings with groups of mothers of children up to five years of age. Although she discusses the importance of giving OPV each time there is an SIA, she also discusses the importance of completing all childhood vaccinations to prevent common childhood diseases (in addition to discussing other health issues like care of the pregnant women, breastfeeding, management of diarrhea through ORS, sanitation and its link with disease, etc). Like IPC meetings, we also expect that successful mothers’ meetings will lead to an increase in both routine and supplemental vaccinations.

### Information education communication (iec) activities used during ipc and mother’s meetings

The CMC is equipped with various IEC materials, including small games, behavioral charts, flip books, flash cards, storytelling, etc., that she uses both at IPC meetings and mothers’ meetings. At every contact (IPC and mothers’ meeting), the CMC assesses perceptions and present behaviors of mothers and, according to their level of understanding, she then discusses the issues and conducts relevant IEC/BCC activities.

In this paper, we examine the performance of routine immunization services, alongside intensive polio eradication efforts in the CGPP areas. We document achievements in access and coverage of routine immunizations and compare these to state-level estimates. Our hypothesis is that CGPP activities to promote routine immunization have helped prevent the potential disruption of routine immunization services by the intensive polio eradication efforts in the same areas. Earlier analyses have explored determinants of the performance of mass campaigns of oral polio vaccine (supplemental immunization activities in addition to routine immunizations) in CGPP areas [13]. In this paper, we investigate the determinants of routine immunization performance. Our additional hypothesis is that the determinants of routine immunization performance---requiring a series of at least seven vaccinations over the first year of life---are likely to be different than determinants of performance of mass campaigns that seek to vaccinate all children under age five once over the course of a few days. Much effort and many resources are being used to strengthen immunization systems in support of polio eradication. Information that can help program managers rationalize which routine immunization promotion activities should be continued, among many, will help improve the efficiency and effectiveness of immunization efforts in UP.

## Methods

### Study design

This study is a secondary analysis of de-identified data originally collected for the purpose of project management. The original data come from CGPP household surveys that include information about the following: vaccination status of children for both routine immunizations and SIAs; mothers’ awareness about routine immunization and SIAs; and exposure of mothers to the social mobilization activities of CGPP India. These data were used to calculate population-based immunization coverage estimates for DPT1, DPT3, and the dropout rate between those who received DPT1 but not DPT3. In the secondary data analysis provided in this paper, we compare findings of three surveys over time and with UP state level estimates from other surveys to judge whether or not there is evidence that routine immunization coverage is worse in areas that have also had an intensive focus on polio eradication. We then used the data from in the latest LQAS survey to model exposure of caretakers to routine immunization promotion activities of the CGPP against receipt of DPT1 and DPT3; this was done to identify possible determinants of better routine immunization coverage.

### Description of data

CGPP India maintains records of project inputs, outputs and some outcomes through a robust management information system (MIS). Thus, all primary beneficiaries of project (children age less than five years) are tracked for vaccination during SIAs and routine immunization sessions. In addition, CGPP India conducted an internal exercise to assess the quality and reach of its social mobilization activities.

The latest project survey in 2011 is the focus of this paper. This latest survey followed principles of lot quality assurance sampling (LQAS) technique. The data were collected in catchment areas of CGPP India, consisting of 10 districts and 56 blocks of Uttar Pradesh. Catchment and supervisory areas (lots) were defined respectively as work areas of District Mobilization Coordinators (DMCs) and Block Mobilization Coordinators (BMCs). The LQAS survey covered a total of 13 catchment areas, consisting of 94 supervision areas. From each supervisory area, a sample of 19 mothers of children less than 12 months of age was selected using a systematic random sampling process. A total of 1,786 face-to-face interviews were conducted by administering a semi-structured interview instrument. The information was collected by BMCs from 25 June 2011 to 24 September 2011. The survey collected the following information: background characteristics of respondent mothers and index children (children aged below 12 months), vaccination status of index children through routine immunization and SIAs, respondent’s awareness about routine immunization and SIAs, recognition of the local CMC, and the respondent mother’s exposure to social mobilization activities of CGPP India---particularly exposure to IPC visits of CMC and mothers’ meeting conducted by CMCs. Survey data was entered and cleaned using MS Excel.

This paper also quotes findings from the reports of prior surveys. Key findings from earlier project baseline and midline surveys are presented for observing the trend of routine immunization coverage in the CGPP catchment areas. Baseline and Midline surveys of CGPP India were conducted by an external agency ‘Synovate’. The data were collected from catchment areas of CGPP India, consisting of 10 districts and 56 blocks of Uttar Pradesh.^1^ Both surveys used a 30-cluster sampling method. The entire catchment area of CGPP India was divided in to two geographical units i.e. 1) Moradabad & Rampur district, and 2) Remaining eight districts. A total of 30 clusters were selected for both the geographical units. A cluster was defined as a village/urban unit or part of a village/ward where CGPP works. Using a structured questionnaire, a total of 10 face-to-face interviews from every cluster were conducted among mothers of children in the age group 12 to 23 months. The information was collected by trained investigators. A total of 605 and 603 interviews were completed for baseline survey and midline survey, respectively. The sample size was selected to provide a 95% confidence interval of plus or minus 5%. The information for baseline survey was collected from 10th July 2008 to 16th July 2008 and for midline survey from 19th July 2010 to 30th July 2010. Both the survey collected following information: profile of respondent mothers, routine immunization coverage, awareness about polio immunization and campaign, awareness of AFP and respondents’ exposure to communication activities of CGPP. The research agency then conducted data entry and analysis and provided the reports of the surveys to the CGPP.

The paper also quotes state level statistics on immunization outcomes. These statistics come from reports of national surveys with disaggregation to the state and/or district level: District level Household Survey- Round 3 (DLSH-3), National Family Health Survey-3 (NFHS-3), a Coverage Evaluation Survey of Uttar Pradesh (CES), and the National Annual Health Survey 2010–11.

### Statistical analysis

For this paper, statistical analysis was only carried out for the latest LQAS survey. Information about other surveys are quoted directly from the survey reports. Routine immunization outcomes for the latest LQAS survey are presented as percentages weighted by the population size of the supervisory areas and with the standard errors adjusted for clustering by supervision area using EPI Info version 7 [14]. The dropout rate for DPT vaccine is computed based on coverage of DPT1 and DPT 3 and presented as percentage. It is computed as “DPT1 coverage – DPT3 coverage / DPT1 coverage * 100”.

To identify the determinants of DPT immunization outcomes (DPT1, DPT3, DPT1-3 Drop Out), we used complex survey analysis programs using STATA (svyset; svy) to conduct exploratory analysis and multivariate logistic regression [15]. This allows for weighting of population size of the blocks (our survey clusters) and adjusts the standard errors for clustering by block. We first used Chi-square tests and univariate logistic regression to identify determinants (our covariates) associated with DPT outcomes. Potential determinants included demographic variables (gender, age of child, religion, education), exposure to program communication events such as mother’s meetings or home visits, and exposure to specific IEC materials and activities (flash cards, snakes and ladder game). Then, in a step-wise approach, we incrementally added potential determinants into the logistic regression model and used likelihood ratio tests to determine the value of each new variable to the fit of the model. Assuming that performance of DPT outcomes would vary by catchment area we also added the catchment area to the model as a categorical variable and assessed significance of the difference in DPT outcomes from a reference catchment area. This also allowed us to calculate the post-estimation prediction of DPT outcomes that varied by catchment area.

## Results

### Description of sample from latest LQAS survey

Table 1 describes the sampling frame of the latest LQAS survey by District, Block and Supervision Area. The survey includes interviews from 1786 mothers of children less than one year of age representing more than 500,000 households in the CGPP catchment area. Tables 2 and 3 provide information about the mothers in the LQAS survey sample. For example, of 1785 mothers who provided information about their religion, 63% were Muslim and 36% were Hindu. The majority of mothers (62% of 1784) reported having no formal education. The mean age of the mothers interviewed was 26.5 years. A description of the children of mothers interviewed in the LQAS survey is provided in Table 4. The mean age of these index children was 6.2 months. The gender breakdown was 52% male, 48% female.

**Table 1:** Sample size covered by district, catchment area and supervision area in Latest LQAS Survey

**District**	**Catchment area (CA)**	**Block name**	**Supervision area (SA)/ BMC area**	**No. of households in SA**	**No. of mothers interviewed**
Baghpat	Baghpat	Baghpat	Baghpat 1	4110	19
			Baghpat 2	4058	19
		Baraut	Baraut 1	4954	19
			Baraut 1	5922	19
		Binauli	Binauli	6360	19
		Chaprauli	Chaprauli	7435	19
		Khekra	Khekra	7918	19
		Pilana	Pilana 1	4437	19
			Pilana 2	4176	19
***Total: CA - Baghpat***				***49370***	***171***
Bareilly	Bareilly	Baheri	Baheri 1	5100	19
			Baheri 2	5125	19
		Bhojipura	Bhojipura	5514	19
		Dalelnagar	Dalelnagar 1	3140	19
			Dalelnagar 1	2630	19
		Meerganj	Meeganj	6817	19
		Nawabganj	Nawabganj 1	4157	19
			Nwabganj 1	4526	19
***Total: CA – Bareilly***				***37009***	***152***
Rampur	Rampur	Bilaspur	Bilaspur 1	5020	19
			Bilaspur 2	6417	19
		Chamrua	Chamrua	5603	19
		Swar	Swar 1	5291	19
			Swar 2	4093	19
		Tanda	Tanda	4514	19
***Total: CA – Rampur***				***30938***	***114***
Meerut	Meerut	Hastinapur	Hastinapur	4147	19
		Kharkhauda	Kharkhauda 1	4134	19
			Kharkhauda 2	3880	19
		Parikshitgarh	Parikshitgarh 1	4694	19
			Parikshitgarh 2	5432	19
		Rohta	Rohta	8034	19
		Sardhana	Sardhana 1	4191	19
			Sardhana 2	4708	19
		Meerut urban	Meerut urban	6803	19
***Total: CA – Meerut***				***46023***	***171***
Moradabad	Moradabad	Zone 4	Zone 4 - 1	6046	19
	(DMC - area 1)		Zone 4 - 2	6532	19
		Bhojpur	Bhojpur 1	6901	19
			Bhojpur 2	6949	19
		Panwasa	Panwasa 1	5095	19
			Panwasa 2	4389	19
***Total : CA – Moradabad (DMC Adnan)***				***35912***	***114***
Moradabad	Moradabad	Zone 3	Zone 3 – 1	5608	19
	(DMC - area 2)		Zone 3 – 2	6079	19
		Munda Pandey	Munda Pandey - 1	5800	19
			Munda Pandey – 2	5052	19
		Naroli	Naroli – 1	5581	19
			Naroli – 2	5491	19
		Manota	Manota – 1	5338	19
			Manota - 2	5654	19
***Total: CA – Moradabad (DMC Anas)***				***44603***	***152***
Moradabad	Moradabad (DMC – area 3)	Zone 5	Zone 5 – 1	5984	19
			Zone 5 – 2	5830	19
			Zone 5 – 3	5943	19
		Sambhal-rural	Sambhal (R) – 1	6770	19
			Sambhal (R) – 2	5339	19
		Sambhal– urban	Sambhal (U) – 1	4060	19
			Sambhal (U)- 2	5652	19
			Sambhal (U)- 3	5877	19
			Sambhal (U) – 4	4378	19
			Sambhal (U) – 5	4718	19
***Total: CA – Moradabad (DMC Sayyad)***				***54551***	***190***
Muzaffar Nagar	Muzaffar Nagar (DMC – area 1)	Budhana	Budhana – 1	5377	19
			Budhana – 2	4229	19
			Budhana – 3	5886	19
			Budhana – 4	3984	19
		Jansath	Jansath – 1	5810	19
			Jansath – 2	6129	19
			Jansath – 3	4933	19
		Khatauli	Khatauli	6258	19
		Purkaji	Purkaji	6915	19
***Total: CA – Muzaffar Nagar (DMC Dushyant)***				***49531***	***171***
Muzaffar Naga	Muzaffar Nagar (DMC – area 2)	Baghra	Baghra	9181	19
		Charthawal	Charthawal 1	5011	19
			Charthawal 2	4907	19
		Shamli	Shamli	7287	19
		Un	Un 1	5246	19
			Un 2	4629	19
***Total: CA – Muzaffar Nagar (DMC Vinod)***				***36261***	***114***
Mau	Mau	Ghosi	Ghosi	10249	19
		Kopaganj	Kopaganj 1	6011	19
			Kopaganj 2	5426	19
		Paradaha	Paradaha	8595	19
		Ranipur	Ranipur	5518	19
***Total: CA – Mau***				***35799***	***95***
Saharanpur	Saharan- pur	Saharanpur City	Saharanpur city 1	7505	19
			Saharanpur city 2	7197	19
		Nakur	Nakur	9086	19
		Sarsawan	Sarsawan	6943	19
		Sunehty	Sunehty 1	6249	19
			Sunehty 2	5388	19
***Total: CA – Saharanpur***				***42368***	***114***
Shahjahan- pur	Shahjahan-pur	Bhawalkheda	Bhawalkheda	7760	19
		Jaitapur	Jaitapur	7840	19
		Kalan	Kalan 1	5434	19
			Kalan 2	6248	19
		Mirzapur	Mirzapur	8105	19
		Sindhuli	Sindhuli	8302	19
***Total: CA – Saharanpur***				***43689***	***114***
Sitapur	Sitapur	Biswan	Biswan	7885	19
		Machhrehata	Machhrehata	8701	19
		Parsendi	Parsendi	7185	19
		Pisawan	Pisawan	9341	19
		Reusa	Reusa	8619	19
		Sanda	Sanda	8311	19
***Total: CA – Sitapur***				***50042***	***114***
**Total : All 13 Catchment Areas (94 Supervision Areas)**				**556096**	**1786**

**Table 2:** Percent distribution of respondent mothers by age, level of education, place of income generation activity and marital status in Latest LQAS Survey

**Characteristics**	Percentage*
**Age (in completed years)**	
17-19 years (%)	02.9
20-24 years (%)	32.8
25-29 years (%)	36.3
30-34 years (%)	17.3
35+ years (%)	10.7
Mean age	26.5
(Number) ^a^	(1780)
**Level of education**	
No formal education (%)	62.2
Primary school (%)	13.2
Middle school (%)	08.6
High school (%)	07.1
Intermediate (%)	03.7
College or above (%)	05.0
Professional education (%)	00.2
(Number) ^a^	(1784)
**Place of income generation activity**	
Work from home - income generation (%)	08.5
Work from outside home - income generation (%)	02.7
No income generation activity (%)	88.8
(Number) ^a^	(1772)
**Marital status**	
Currently married (%)	99.6
Widowed/ Divorced/ Separated (%)	00.4
(Number) ^a^	(1786)

* Percentages are weighted by population of supervisory areas.

^a^ Number of interviews without missing information.

**Table 3:** Percent distribution of respondent mothers by number of surviving children, years of stay in the same community, and religion in Latest LQAS Survey

Characteristics*	Percentage*
**Total number of surviving children**	
1 child (%)	23.4
2 children (%)	23.9
3 children (%)	19.5
4 children (%)	11.3
5 children (%)	09.6
6 or more children (%)	12.3
Mean number of surviving children	3.09
(Number) ^a^	(1782)
**Years of stay in the same community (village/ward)**	
Below 1 year	01.5
1-2 years	16.9
3-4 years	19.5
5+ years	62.1
Median years of stay	7.8
(Number) ^a^	(1724)
**Religion**	
Hindu (%)	36.0
Muslim (%)	63.4
Others ^b^	00.6
(Number) ^a^	(1785)

* Percentages are weighted by population of supervisory areas.

^a^ Number of interviews without missing information.

^b^ Includes - Christian, Sikh, Jain, etc.

**Table 4:** Percent distribution of index children by sex, age and place of birth in Latest LQAS Survey

Characteristics*	Percentage*
**Sex**	
Male (%)	52.0
Female (%)	48.0
(Number) ^a^	(1786)
**Age (in completed months)**	
< 1 month (%)	07.0
1 month (%)	09.0
2 months (%)	07.0
3 months (%)	07.5
4 months (%)	06.9
5 months (%)	07.9
6 months (%)	09.3
7 months (%)	09.6
8 months (%)	09.2
9 months (%)	08.2
10 months (%)	09.6
11 months (%)	08.8
Mean age	6.21
(Number) ^a^	(1786)
**Place of birth**	
Institution (%)	54.7
Home or others (%)	45.3
(Number) ^a^	(1762)

* Percentages are weighted by population of supervisory areas.

^a^ Number of interviews without missing information.

### Routine immunization performance

Table 5 shows key results from three project surveys in 2008, 2010 and 2011. The surveys in 2008 and 2010 provide information about vaccinations received by children 12–23 months of age. The latest LQAS survey in 2011 provides information about vaccines received by children less than 12 months and provides a breakdown by several different age groups (10–11 months, 11 months) that most closely compare with the 2007 and 2010 surveys. The proportion of children who received BCG, 3 doses of DPT, 3 doses of OPV and measles vaccine (fully immunized children) has increased from 48% in Baseline (2007) to 64% in Midline Survey (2010). The LQAS survey conducted in 2011 among children aged below 12 months also indicates improvement in the RI coverage compared to the baseline survey. It shows that more than two-thirds of children are vaccinated at the right time for primary antigen (BCG to Measles). Since the baseline survey, the dropout rate for DPT vaccine has decreased. These improvements in routine immunization performance occurred among children living in communities with intensive polio eradication efforts occurring contemporaneously. This would not be expected if routine immunization services were being disrupted by polio eradication efforts.

**Table 5:** Trend in Routine Immunization performance in the catchment area of CGPP India, 2007-2011

**CGPP/India survey**	**Age group of index children**	**Survey year**	% Children received (95% CI)**		**% Drop out (DPT1-DPT3)**	% Fully immunized children*	**N**
			**DPT1**	**DPT3**			
Baseline (30 cluster)	12-23 months	2008	86.5	71.4	17.5	48.1	185
Midline (30 cluster)	12-23 months	2010	93.5	81.1	13.3	63.8	185
Latest (LQAS)	10-11 months	2011	94.2 (90.6-97.8)	81.3 (76.3-86.2)	13.7	55.2 (48.6-61.7)	286
	11 months		92.7 (87.0-98.5)	84.1 (77.3-90.8)	09.3	62.7 (53.2-72.1)	135

* Children received BCG, 3 DPTs, 3 OPVs and measles vaccine.

** Confidence intervals presented where available.

Table 6 provides UP state level estimates of routine immunization coverage from several national health surveys. The key findings from these surveys indicate that RI coverage has increased over time also. A nation-wide Annual Health Survey in 2010–11 reports that about 45% children were fully immunized in Uttar Pradesh compared with about 20% when the National Family Health Survey 3 was done in 2005–06. All during this period, Uttar Pradesh was participating in intensive polio eradication efforts. The CGPP project provided additional polio eradication inputs since 2003---primarily social mobilization---above and beyond the efforts of the UP Ministry of Health and Family Welfare in the CGPP catchment area. If routine immunization services were disrupted due to polio eradication efforts, then it would be expected that routine immunization coverage might be worse in CGPP areas as compared to the UP state averages. However, the levels of coverage in CGPP areas have increased over time and have remained higher than reported state levels throughout this period. Looking at the findings across Tables 5 and 6, there is no evidence that areas with intensive polio eradication efforts are disrupting routine immunization services.

**Table 6:** Trend in Routine Immunization performance among children aged 12–23 months in Uttar Pradesh, India 2007-2011

**Data source**	**Survey year**	**% Children received**		**% Drop out (DPT1-DPT3)**	% Fully immunized children*	**N**
		**DPT1**	**DPT3**			
National Family Health Survey-3, UP	2005-06	55.7	30.0	46.1	20.3	1364
District level Household Survey-3, UP	2007-08	65.4	38.8	40.7	30.2	12141
Coverage Evaluation Survey, UP	2009	73.4	58.1	20.8	40.9	1112
Annual Health Survey, UP**	2010-11	--	55.9	--	45.3	75332***

* Children received BCG, 3 DPTs, 3 OPVs and measles vaccine.

** Annual Health Survey 2010–11 Fact Sheet (full report not available to authors).

*** Number derived from percent of children 12–23 months of age with immunization card.

### Determinants of routine immunization performance

Information about the relationship and interaction between the mothers interviewed in the latest LQAS survey and their assigned CMC is presented in Table 7. Almost all mothers surveyed (96.9) know their assigned CMC, and a majority (64.8%) know their CMC by their designation as a CMC. Almost all mothers (94%) report that the CMC had visited their home within the three months prior to the survey. And, many mothers (30.2%) reported having attended a mother’s meeting---organized their CMC---within the three months prior to the survey.

**Table 7:** Percent distribution of respondent mothers by recognition of CMC, exposure to home visits of CMC and exposure to mothers meeting

**Information**	Percentage*
Percent respondents recognise CMC by designation*	64.8
(Number) ^a^	(1779)
Percent respondents recognise CMC by name or designation or any other identity*	96.9
(Number) ^a^	(1783)
Percent respondents stated that CMC visited home in the last three months*	94.0
(Number) ^a^	(1783)
Percent respondents attended/participated in a mothers’ meeting organised by CMC in the last three months*	30.2
(Number) ^a^	(1777)

* Percentages are weighted by population of supervisory areas.

^a^ Number of interviews without missing information.

Exposure to specific IEC materials---provided by CMCs---among mothers interviewed in the latest LQAS survey is summarized in Table 8. Of the 1786 mothers interviewed, 78.4% reported exposure to at least one IEC material provided by CGPP CMCs. The IEC materials that mothers most frequently reported they had exposure to include the following: congratulatory card (*Badhai)* cards (59%); leaflets on routine immunization (49%); and, flash cards (32%). The least frequently mentioned IEC exposures were the snakes and ladder game (3%), flip books (*Khoji Amma*, 7%), and behavior charts (13%).

**Table 8:** Percent distribution of respondent mothers by exposure status to IEC materials developed by CGPP India

**Exposure status**	Percentage*
Exposed to a **‘leaflet on RI’** during IPC home visits of CMC or mothers’ meeting (%)	48.8
(Number)^a^	(1781)
Exposed to a **flip book ( *Khoji Amma *)** during IPC home visits of CMC or mothers’ meeting (%)	07.4
(Number)^a^	(1781)
Exposed to **CMC message kit *(CMC ki potli)*** during IPC home visits of CMC or mothers’ meeting (%)	21.7
(Number)^a^	(1781)
Exposed to **‘flash cards’** during IPC home visits of CMC or mothers’ meeting (%)	31.6
(Number)^a^	(1781)
Exposed to a **congratulatory card *(Badhai card)*** during IPC home visits of CMC (%)	59.4
(Number)^a^	(1764)
Exposed to **‘behaviour charts’** during mothers’ meeting (%)	13.2
(Number)^a^	(1767)
Exposed to ‘**snakes and ladders game**’ during mothers’ meeting (%)	03.1
(Number)^a^	(1767)
Exposed to **at least one IEC material of CGPP India** during IPC home visits of CMC or mothers’ meeting (%).	78.4
(Number)	(1786)

* Percentages are weighted by population of supervisory areas.

^a^ Number of interviews without missing information.

Table 9 shows the results of the multivariate logistic regression analysis of determinants of DPT1 coverage. Three determinants were identified. Having a formal education or non-Muslim religion predicts an increased DPT1 coverage of 6%. Mother’s exposure to IEC flash cards with information about routine immunization predicts an increase in DPT1 coverage of about 4%. DPT1 coverage in Saharanpur was 1% to 7% higher than all other catchment areas. Table 10 shows the regression model’s predicted DPT1 coverage by catchment area and by various combinations of determinants. DPT1 coverage is predicted to be lowest (84%) in the Moradabad 3 catchment area under the following conditions: mothers are Muslim, have no formal education, and are not exposed to an IEC flash card during a mother’s meeting or home visit. Predicted DPT1 coverage, however, rises to 100% in all catchment areas under the opposite conditions (all mothers are not Muslim, all have formal education, and all are exposed to IEC flash cards). Among all families, DPT1 coverage is predicted to increase 9% if the mother has a formal education and exposure to IEC flash cards.

**Table 9:** Determinants of DPT1 coverage among children 6–11 months of age in LQAS survey*

**Variable**	**Coefficient**	**Std. Err.**	**z**	**p value**	**[95% Confidence interval]**	
(Constant)	0.918804	0.018823	48.81	0.000	0.881426	0.956183
Mother has formal education	0.058082	0.014099	4.12	0.000	0.030083	0.086079
Mother’s religion other than Muslim	0.059198	0.012428	4.76	0.000	0.034518	0.083878
Mother exposed to IEC flash card	0.043343	0.013593	3.19	0.002	0.01635	0.070336
Catchment Area						
Saharanpur (reference )	--	--	--	--	--	--
Bareilly	−0.00620	0.02573	−0.24	0.81	−0.0573	0.044891
Rampur	−0.00769	0.033696	−0.23	0.82	−0.07461	0.059222
Meerut	−0.05703	0.022926	−2.49	0.015	−0.10256	−0.01151
Moradabad1	−0.03753	0.027922	−1.34	0.182	−0.09298	0.017915
Moradabad2	−0.05293	0.050749	−1.04	0.3	−0.15371	0.047844
Moradabad3	−0.07642	0.03222	−2.37	0.02	−0.1404	−0.01243
Muzaffarnagar1	−0.02557	0.03539	−0.72	0.472	−0.09584	0.044712
Muzaffarnagar2	−0.06842	0.034915	−1.96	0.053	−0.13775	0.000913
Mau	−0.01327	0.019583	−0.68	0.5	−0.05216	0.025616
Baghpat	−0.05644	0.027325	−2.07	0.042	−0.1107	−0.00218
Shahjahanpur	−0.04419	0.03387	−1.3	0.195	−0.11144	0.023073
Sitapur	−0.05025	0.022627	−2.22	0.029	−0.09518	−0.00532

* Analyses are weighted by the population size of blocks and the standard errors are adjusted for clustering by block.

**Table 10:** Predicted DPT1 coverage by District among children 6–11 months of age in LQAS survey*

	**Multivariate analysis determinants**			
**Catchment area**	**Index (Religion Muslim, No Formal Education, Mother does not exposed to IEC flash card)**	**Religion other than Muslim**	**Religion other than Muslim plus Formal Education**	**Religion other than Muslim plus Formal Education plus Mother exposed to IEC flash card**
Saharanpur	92%	98%	>100%	>100%
Bareilly	91%	97%	>100%	>100%
Rampur	91%	97%	>100%	>100%
Meerut	86%	92%	98%	>100%
Moradabad1	88%	94%	100%	>100%
Moradabad2	87%	93%	98%	>100%
Moradabad3	84%	90%	96%	100%
Muzaffarnagar1	89%	95%	>100%	>100%
Muzaffarnagar2	85%	91%	97%	>100%
Mau	91%	96%	>100%	>100%
Baghpat	86%	92%	98%	>100%
Shahjahanpur	87%	93%	99%	>100%
Sitapur	87%	93%	99%	>100%

* Predictions are based on post-estimation linear combinations of estimates in model in Table 9 above. These predictions are adjusted for clustering within the Block where children live and weighted by the estimated number of households in the Block.

Table 11 shows the results of the multivariate logistic regression analysis of determinants of DPT3 coverage. Three determinants were identified, one different from the DPT1 determinants. Having a formal education or non-Muslim religion predicts an increased DPT3 coverage of 12% and 13%, respectively. Mother’s recognition of her local CMC by designation predicts an increase in DPT3 coverage of about 12%. DPT3 coverage in Saharanpur was 16% to 36% higher than all other catchment areas. Table 12 shows the regression model’s predicted DPT3 coverage by catchment area and by various combinations of determinants. DPT3 coverage is predicted to be lowest (35%) in the Moradabad 3 catchment area under the following conditions: all mothers are Muslim, have no formal education, and do not know their local CMC by designation. Predicted DPT3 coverage, however, rises by 38% in all catchment areas under the opposite conditions (all mothers are not Muslim, all have formal education, and all know their local CMC by designation); for example, in the Moradabad 3 catchment area, DPT3 coverage would be predicted to rise from 35% to 73% under these conditions. Among all families, DPT3 coverage is predicted to increase 25% if the mothers have a formal education and know their local CMC by designation.

**Table 11:** Determinants of DPT3 coverage among children 6–11 months of age in LQAS survey*

**Variable**	**Coefficient**	**Std. Err.**	**z**	**p value**	**[95% Confidence interval]**	
(Constant)	.7092458	.0694324	10.21	0.000	.5713669	.8471247
Mother has formal education	.1245145	.0360619	3.45	0.001	.0529027	.1961263
Mother’s religion other than Muslim	.1311864	.0326796	4.01	0.000	.0662912	.1960817
Mother recognizes CMC by designation	.1244052	.036497	3.41	0.001	.0519294	.196881
Catchment Area						
Saharanpur (reference )	--	--	--	--	--	--
Bareilly	-.1632748	.0849561	−1.92	0.058	-.3319807	.0054312
Rampur	-.1917798	.11941	−1.61	0.112	-.4289043	.0453447
Meerut	-.1875548	.0798418	−2.35	0.021	-.3461048	-.0290047
Moradabad1	-.2233971	.1068674	−2.09	0.039	-.4356146	-.0111797
Moradabad2	−0.28396	0.112693	−2.52	0.013	−0.50775	−0.06018
Moradabad3	−0.36005	0.069865	−5.15	0.000	−0.49879	−0.22132
Muzaffarnagar1	−0.19526	0.089303	−2.19	0.031	−0.3726	−0.01792
Muzaffarnagar2	−0.27419	0.069381	−3.95	0.000	−0.41197	−0.13641
Mau	−0.23171	0.127447	−1.82	0.072	−0.48479	0.021376
Baghpat	−0.24329	0.073031	−3.33	0.001	−0.38831	−0.09826
Shahjahanpur	−0.34274	0.097671	−3.51	0.001	−0.53669	−0.14878
Sitapur	−0.24175	0.068834	−3.51	0.001	−0.37844	−0.10506

* Analyses are weighted by the population size of blocks and the standard errors are adjusted for clustering by block.

**Table 12:** Predicted DPT3 coverage by District among children 6–11 months of age in LQAS survey*

	**Multivariate analysis determinants**			
**Catchment area**	**Index (Religion Muslim, No Formal Education, Mother does not know CMC by designation)**	**Religion other than Muslim**	**Religion other than Muslim plus Formal Education**	**Religion other than Muslim plus Formal Education plus Mother Knows CMC by designation**
Saharanpur	71%	84%	96%	>100%
Bareilly	55%	68%	80%	93%
Rampur	52%	65%	77%	90%
Meerut	52%	65%	78%	90%
Moradabad1	49%	62%	74%	87%
Moradabad2	43%	56%	68%	81%
Moradabad3	35%	48%	60%	73%
Muzaffarnagar1	51%	65%	77%	89%
Muzaffarnagar2	44%	57%	69%	82%
Mau	48%	61%	73%	86%
Baghpat	47%	60%	72%	85%
Shahjahanpur	37%	50%	62%	75%
Sitapur	47%	60%	72%	85%

* Predictions are based on post-estimation linear combinations of estimates in model in Table 11 above. These predictions are adjusted for the Block where children live and weighted by the estimated number of households in the Block.

The results of the analysis of determinants of the DPT1-3 Drop Out Rate are shown in Table 13. The same three determinants for DPT3 are relevant also to the Drop Out Rate. Having either a formal education or non-Muslim religion predicts a decrease in the Drop Out Rate of about 10%. Mother’s recognition of her local CMC by designation predicts a decrease in the DPT1-3 Drop Out Rate of about 11%. The Drop Out Rate in Saharanpur (24%) was 15% to 33% lower than all other catchment areas. Table 14 shows the regression model’s predicted Drop Out Rate by catchment area and by various combinations of determinants. The Drop Out Rate is predicted to be highest in the Moradabad 3 and Shahjahanpur catchment areas (57% and 56%, respectively) under the following conditions: all mothers are Muslim, have no formal education, and do not know their local CMC by designation. The predicted Drop Out Rate, however, decreases by 30% in all catchment areas under the opposite conditions (all mothers are not Muslim, all have formal education, and all know their local CMC by designation); for example, in the Moradabad 3 catchment area, the Drop Out Rate would be predicted to decrease from 57% to 27% under these conditions. Among all families, the DPT1-3 Drop Out Rate is predicted to decrease by 18% if the mothers have a formal education and know their local CMC by designation.

**Table 13:** Determinants of DPT Drop Out Rates among children 6–11 months of age in LQAS survey*

**Variable**	**Coefficient**	**Std. Err.**	**z**	**p value**	**[95% Confidence interval]**	
(Constant)	0.241152	0.061777	3.9	0.000	0.118475	0.363828
Mother has formal education	−0.09611	0.03539	−2.72	0.008	−0.16639	−0.02583
Mother’s religion other than Muslim	−0.09696	0.033258	−2.92	0.004	−0.163	−0.03091
Mother recognizes CMC by designation	−0.10669	0.03666	−2.91	0.005	−0.17949	−0.03389
Catchment Area						
Saharanpur (reference)	--	--	--	--	--	--
Bareilly	0.154367	0.076535	2.02	0.047	0.002384	0.30635
Rampur	0.18252	0.101189	1.8	0.075	−0.01842	0.383461
Meerut	0.151472	0.072155	2.1	0.039	0.008186	0.294758
Moradabad1	0.202337	0.101167	2	0.048	0.00144	0.403235
Moradabad2	0.253376	0.101143	2.51	0.014	0.052526	0.454227
Moradabad3	0.327629	0.068603	4.78	0.000	0.191398	0.46386
Muzaffarnagar1	0.174604	0.078216	2.23	0.028	0.019283	0.329924
Muzaffarnagar2	0.238405	0.068737	3.47	0.001	0.101907	0.374902
Mau	0.228856	0.128518	1.78	0.078	−0.02635	0.484067
Baghpat	0.208591	0.06784	3.07	0.003	0.073873	0.343308
Shahjahanpur	0.316697	0.095478	3.32	0.001	0.127097	0.506298
Sitapur	0.209165	0.062604	3.34	0.001	0.084846	0.333485

* Analyses are weighted by the population size of blocks and the standard errors are adjusted for clustering by block.

**Table 14:** Predicted DPT Drop Out Rates by District among children 6–11 months of age in LQAS survey*

	**Multivariate analysis determinants**			
**Catchment area**	**Index (Religion Muslim, No Formal Education, Mother does not know CMC by designation)**	**Religion other than Muslim**	**Religion other than Muslim plus Formal Education**	**Religion other than Muslim plus Formal Education plus Mother Knows CMC by designation**
Saharanpur	24%	14%	5%	<0%
Bareilly	40%	30%	20%	10%
Rampur	42%	33%	23%	12%
Meerut	39%	30%	20%	9%
Moradabad1	44%	35%	25%	14%
Moradabad2	49%	40%	30%	19%
Moradabad3	57%	47%	38%	27%
Muzaffarnagar1	42%	32%	22%	12%
Muzaffarnagar2	48%	38%	29%	18%
Mau	47%	37%	28%	17%
Baghpat	45%	35%	26%	15%
Shahjahanpur	56%	46%	36%	26%
Sitapur	45%	35%	26%	15%

* Predictions are based on post-estimation linear combinations of estimates in model in Table 13 above. These predictions are adjusted for the Block where children live and weighted by the estimated number of households in the Block.

## Discussion

### Limitations

The main limitation of the analysis is that inferences rely on observational and cross sectional data. A randomized controlled trial testing CGPP interventions for effects on routine immunization was not possible. Where possible, the results of the latest LQAS survey were compared with similar data from earlier time periods and state level estimates from other sources. However, identification of the counterfactual was not possible. In addition, detection of differences over time or between the program area and state averages, through statistical tests with defined levels of power, was not possible. Inferences regarding determinants of routine immunization coverage are therefore based solely upon statistical association of routine immunization performance indicators with exposure to IEC materials and other respondent factors. Another limitation of the analysis is that exposure to IEC materials does not include information about the quality of the exposure but only about the absence or presence of the exposure.

### Routine immunization performance

If intensive polio eradication efforts were detrimental to routine immunization, we might expect no improvement or worsening levels of immunization coverage in the presence of these efforts. However, immunization coverage has improved considerably over time at the state level and in CGPP areas. This occurred during a period of intense polio eradication efforts as evidenced with the interruption of polio transmission by the end of 2011. In addition, immunization coverage appears higher in CGPP program areas compared to state averages, even though these areas have a level of intensity of polio eradication efforts greater than in the rest of the state. See Weiss et al. (2011 & 2013) for more details about the polio eradication activities of the CGPP [12,13]. It is possible that routine immunization coverage would have improved more without these polio eradication efforts, but this hypothesis is not testable. At minimum, there is no evidence that immunization coverage was disrupted to the point that routine immunization became worse in the presence of polio eradication efforts at either the state level or within CGPP areas. And, there is no evidence that routine immunization in CGPP areas was worse than performance at the state level despite increased intensity of polio eradication efforts in these areas. In CGPP areas, special attention was made to strengthen the routine immunization systems alongside of polio eradication efforts (e.g., promote routine immunizations alongside CMC activities to promote polio vaccination during mass campaigns) and these data appear to support the continuation of these system strengthening activities. The specific activities of the CGPP that are most helpful in supporting routine immunization are discussed below.

### Determinants of routine immunization performance

A mother’s background (religion and education status) is a determinant of DPT immunization outcomes. However, religion is not a target of change, and improving education status on a population level will require a long-term effort. To address these two determinants, programs need to tailor their strategies such as promoting immunization through religious leaders or use IEC materials better suited for illiterate populations.

Several determinants more sensitive to change by program efforts were identified. DPT1, a measure of access to immunization, was affected by exposure of caretakers to IEC materials that promoted routine immunizations, and that were provided by CMCs at the same time the CMCs were promoting participation in mass polio vaccination campaigns. Specifically, exposure to flash cards promoting routine immunization during mother’s meetings or home visits by CMCs predicts an increase in DPT1 coverage of about 4%; these mother’s meetings and home visits were a key strategy to encourage caretakers to have their children vaccinated with polio during an upcoming mass campaign. To improve DPT3 coverage and lower DPT Drop Out rates, a situation where mothers of infants know their local CMC is helpful. This suggests that while a simple intervention (flash cards at a mother’s meeting or home visit) can improve coverage of the first DPT vaccination, a longer-term, personal relationship with local health workers in this setting is what helps improve coverage of a complete series of vaccinations by avoiding a large number of drop outs between the first and last vaccination.

Interestingly, recent attendance at mother’s meeting or having a recent home visit by a CMC were not associated with routine immunization---when the analysis was adjusted for the mothers’ background variables or relevant program activities. This suggests that it is the content of the meetings (e.g., IEC activities like flash cards), not the meetings themselves, that are more important for increasing access (e.g., DPT1) to immunization. In addition, having a longer term relationship with the CMC, across many meetings or visits, appears more important for increasing DPT3 coverage or reducing Drop-Out Rates than exposure to a recent meeting or to specific IEC materials.

## Conclusions

In the CGPP catchment areas, intensive polio eradication activities did not appear to disrupt routine immunization coverage. Routine immunization can be promoted alongside polio eradication efforts. CGPP provided IEC materials and messages promoting routine immunization during social mobilization activities carried out to encourage caretakers to have their children vaccinated during polio mass campaigns. While IEC activities were helpful in increasing access to routine immunizations, IEC activities do not appear sufficient to achieve high levels of routine immunization coverage. Longer term relationships, between caretakers and local health workers who are supportive of childhood immunizations, appear more important and may reflect issues of trust in the health system. Strategies for promotion of immunization in this setting need to be tailored to the religious and education background of caretakers.

### Consent

Verbal consent was obtained from the child’s caretaker prior to administration of the LQAS survey. The LQAS survey was carried out for the purpose of project management and not research. The authors of this report later conducted secondary data analysis of de-identified survey data and did not have access to personal identifiers of the survey respondents or have any further contact with survey respondents for the secondary data analysis.

## Endnotes

^a^Two districts, Mordabad and Muzaffarnagar, were each divided into two districts for a total of 12 districts instead of 10.

## Competing interests

All authors have received salary support from the US Agency for International Development (USAID) under Cooperative Agreement GHN-A-00-07-00014. This salary support has covered implementation of the project described and/or for writing this manuscript.

## Authors’ contribution

WW and MC wrote key sections of the Background, Methods, Results, Discussions and Conclusions. WW and MC also designed and carried out exploratory and statistical analysis. RS wrote key sections of the Background, Methods and edited the manuscript. All authors have read and approved the final version of the manuscript.

## Pre-publication history

The pre-publication history for this paper can be accessed here:

http://www.biomedcentral.com/1472-698X/13/25/prepub
